# Markerless Motion Capture to Quantify Functional Performance in Neurodegeneration: Systematic Review

**DOI:** 10.2196/52582

**Published:** 2024-08-06

**Authors:** Julian Jeyasingh-Jacob, Mark Crook-Rumsey, Harshvi Shah, Theresita Joseph, Subati Abulikemu, Sarah Daniels, David J Sharp, Shlomi Haar

**Affiliations:** 1 Department of Brain Sciences Imperial College London London United Kingdom; 2 Care Research and Technology Centre UK Dementia Research Institute Imperial College London London United Kingdom; 3 Department of Basic and Clinical Neuroscience King's College London London United Kingdom

**Keywords:** markerless motion capture, motion analysis, movement analysis, motion, neurodegeneration, neurodegenerative, systematic review, movement, body tracking, tracking, monitoring, clinical decision making, decision, decision making, dementia, neurodegenerative disease, mild cognitive impairment, Parkinson's disease, tool, mobility

## Abstract

**Background:**

Markerless motion capture (MMC) uses video cameras or depth sensors for full body tracking and presents a promising approach for objectively and unobtrusively monitoring functional performance within community settings, to aid clinical decision-making in neurodegenerative diseases such as dementia.

**Objective:**

The primary objective of this systematic review was to investigate the application of MMC using full-body tracking, to quantify functional performance in people with dementia, mild cognitive impairment, and Parkinson disease.

**Methods:**

A systematic search of the Embase, MEDLINE, CINAHL, and Scopus databases was conducted between November 2022 and February 2023, which yielded a total of 1595 results. The inclusion criteria were MMC and full-body tracking. A total of 157 studies were included for full-text screening, out of which 26 eligible studies that met the selection criteria were included in the review.

**Results:**

Primarily, the selected studies focused on gait analysis (n=24), while other functional tasks, such as sit to stand (n=5) and stepping in place (n=1), were also explored. However, activities of daily living were not evaluated in any of the included studies. MMC models varied across the studies, encompassing depth cameras (n=18) versus standard video cameras (n=5) or mobile phone cameras (n=2) with postprocessing using deep learning models. However, only 6 studies conducted rigorous comparisons with established gold-standard motion capture models.

**Conclusions:**

Despite its potential as an effective tool for analyzing movement and posture in individuals with dementia, mild cognitive impairment, and Parkinson disease, further research is required to establish the clinical usefulness of MMC in quantifying mobility and functional performance in the real world.

## Introduction

Markerless motion capture (MMC) technology uses sensors and advanced software algorithms to track and analyze human movement, without the attachment of physical markers to individuals or the use of external devices such as pressure sensors or wearables. There is growing use of MMC to provide highly accurate quantitative parameters of physical function including mobility [1,2], balance [3], upper extremity tasks [4], and activities of daily living (ADL) [5].

While 3D motion capture systems using markers are considered the gold standard for movement analysis, they have several limitations including their lack of portability, the need for trained staff, and the requirement for reflective markers to be placed precisely on participants’ bodies [6]. In contrast, the use of MMC provides several advantages: being easier to operate, requiring less space, and being more economical than traditional marker-based systems [7]. Importantly, their ability to capture movement unobtrusively is a key benefit for user compliance [8], particularly when working with individuals with cognitive impairments.

MMC is attractive for health care and research use, such as monitoring functional performance loss or improvement in neurodegenerative diseases. While traditional movement analyses are based on subjective clinical assessments, MMC can be used to generate objective and quantifiable digital biomarkers that can help detect a decline in functional performance by capturing movement unobtrusively [9]. Variations in these digital biomarkers could indicate underlying impairment and enable earlier support. The fact that MMC can be deployed in home environments may avoid unnecessary hospital visits for patients, as well as detect subtle changes in functional ability that may only be apparent in everyday home-based settings rather than within a clinic.

Several MMC devices can provide cost-effective assessments of functional performance in research and clinical settings. Broadly, the 2 main types of MMC camera hardware are depth cameras and standard red-green-blue (RGB) video cameras, used in single or multicamera systems. Commonly used and widely accessible depth cameras are the Kinect (Microsoft) devices, which use standard RGB color video as well as depth estimation by recording the distance between the camera and each pixel through the emission of structured light patterns [10]. Machine learning algorithms can be used to reconstruct 3D skeletal models in real-time from the RBG+depth (RBG-D) image. Alternatively, deep learning can be used with standard video cameras or mobile phone cameras to record limb location and orientation. This method uses deep neural networks trained from large datasets to estimate body segment position and orientation (pose) and motion tracking, without explicit depth sensing. It requires specific body segment positions known as the 6 degrees of freedom: 3 rotational (flexion or extension, abduction or adduction, and rotation about the longitudinal axis) and 3 translational (sagittal, frontal, and transverse) [11]. Both forms of MMC have shown promising use thus far.

A scoping review of single-camera MMC models used in health care highlighted the significant potential for use in clinical applications but also noted the need to improve their tracking accuracy [12]. A previous systematic review of MMC-based training devices used in neurological rehabilitation found that these devices improve motivation and enable better functional performance potentially due to the gaming element [13]. Another systematic review of MMC-based devices in rehabilitation found that balance training with the support of MMC resulted in better outcomes potentially due to more dynamic training conditions [14]. While those systematic reviews explored the use of MMC specifically in rehabilitation training, this review focuses on the technology-based evaluation of functional tasks. The recent increase in the number of studies involving MMC-based movement analysis in neurodegenerative diseases offers a strong rationale for this review. This trend includes the use of MMC to track gait decline [9], assess fall risks [15,16], detect disease traits [17], estimate disease severity [18], and detect cognitive impairment from gait features [19].

Neurodegenerative diseases such as dementia and Parkinson disease (PD) lead to declining functional performance. Detecting problems in everyday functional tasks in these patient groups can help provide early, timely, and clinically appropriate interventions that may help maintain independence, decrease caregiver burden, and potentially slow the rate of functional decline [20,21]. MMC can provide digitally measured functional performance data that could be used to enhance clinical decision-making and remote monitoring; identify risks such as falls; and better capture the impact of rehabilitative, pharmacological, and surgical interventions. Although MMC technology could offer the potential for detecting functional changes in neurodegenerative diseases, a model that is comparable to established gold-standard motion capture systems is essential for deployment in real-world applications. This study aimed to complete a systematic review of published literature on the use of MMC with full-body tracking for quantifying functional performance in people with dementia, mild cognitive impairment (MCI), and PD.

## Methods

### Study Design

The web-based Covidence (Veritas Health Innovation) software platform was used in this review, and the titles and abstracts were screened by 2 independent reviewers. The full text of the relevant studies was reviewed, and the quality of the studies was assessed by 2 independent reviewers. Data extraction was also performed by 2 independent reviewers, and any conflicts were resolved through discussion.

### Search Strategy

The search strategy was designed to include all types of studies that used MMC with full-body tracking in individuals with dementia, MCI, or PD. To identify relevant studies, a combination of both the Medical Subject Headings thesaurus and free-text terms related to the 3 conditions and MMC technology were used. The search included publications from all years in the CINAHL, Embase, MEDLINE, and Scopus databases using the terms “Motion Capture,” “Motion Analysis,” “Movement Analysis,” and “Pose Estimation” in combination with “Dementia,” “Mild cognitive impairment,” and “Parkinson’s disease.” The details of the search activity can be found in Multimedia Appendix 1.

### Inclusion and Exclusion Criteria

The inclusion criteria for the systematic review were as follows: (1) markerless optical motion capture; (2) full-body tracking; (3) involving participants with dementia, MCI, or PD; (4) original research; and (5) English language studies. Studies with the following characteristics were excluded: (1) motion capture with markers, inertial measurement units, body-worn sensors, or pressure sensors; (2) movement analysis of specific parts of the body or symptoms such as tremor and rigidity; (3) evaluating interventions such as exercises, deep brain stimulation, medication, rehabilitation protocol, dance, and gaming; and (4) pose estimation of videos found on the internet.

### Data Extraction

The general information extracted from the studies included: the center and country where the study took place; study characteristics; funding sources; age, sex, and number of participants; number and duration of visits; study aims; inclusion and exclusion criteria; and the main disease condition evaluated. Methodological information extracted included technical details of the MMC system used; functional performance area evaluated, for example, gait or sit to stand; software used for feature extraction; and the method of analysis. The results information extracted included the following: statistically significant movement features, whether they were measured under single or dual task (motor or cognitive) conditions, whether compared to established gold standard models or a relevant clinical measure, and key outcomes including the level of accuracy obtained.

## Results

### Study Selection

**Figure 1:**
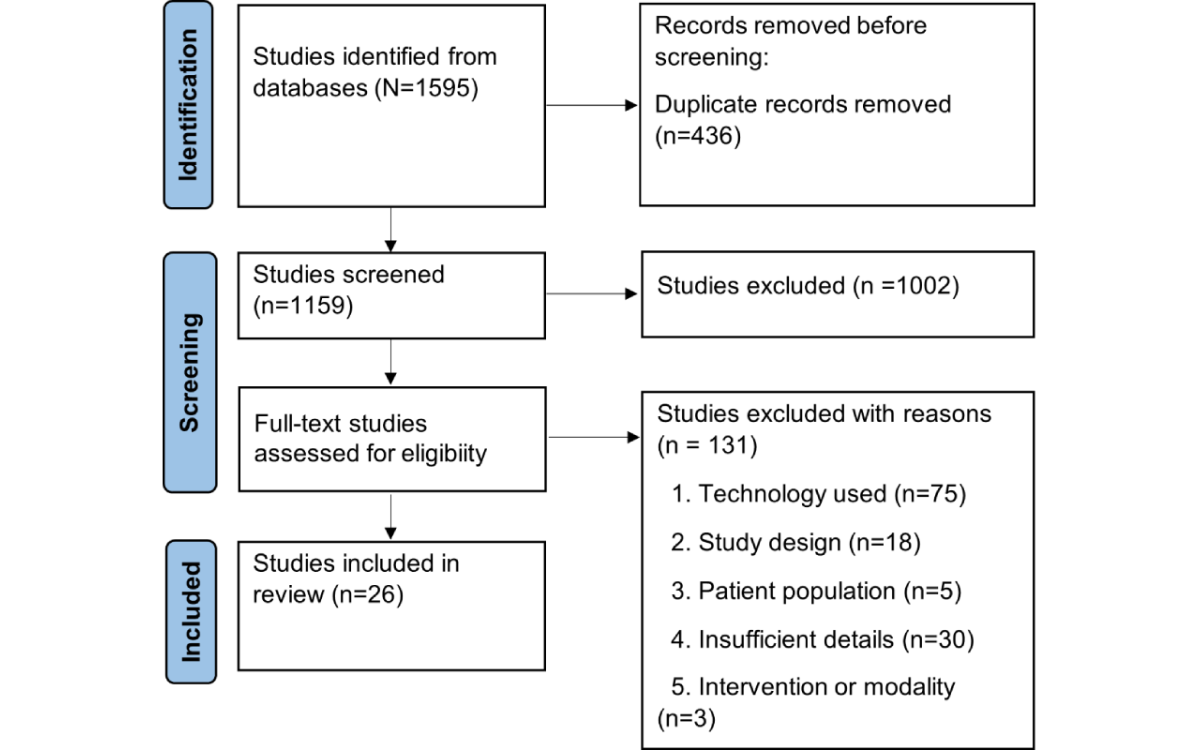
PRISMA (Preferred Reporting Items for Systematic Reviews and Meta-Analyses) 2020 flow diagram for new systematic reviews.

The literature search yielded 1595 results; after removing duplicates, 1159 studies remained for title or abstract screening. Subsequently, 131 studies were identified for full-text screening, of which 26 studies met the inclusion criteria and were included in the review. A PRISMA (Preferred Reporting Items for Systematic Reviews and Meta-Analyses) flowchart [22] outlining the selection process can be found in Figure 1.

### Quality Assessment

The 26 selected studies were assessed for quality using the Specialist Unit for Review Evidence questions to assist with the critical appraisal of the cross-sectional studies tool [23]. While no studies were excluded from the review based on this assessment, issues pertaining to quality were identified within several of the studies. Table 1 shows that all studies included in this review used appropriate outcome measures (n=26, 100%). Most studies clearly stated the study design (n=20, 77%); provided information on the study setting, location, and dates (n=20, 77%); described the results well (n=19, 73%); and provided participant characteristics (n=16, 62%). However, few studies reported if participants were fairly selected (n=10, 38%) or provided information on participant eligibility (n=2, 8%) and handling of missing data and control of potential biases (n=1, 4%).

**Table 1:** Quality assessment summary.

Study	Is the study design clearly stated?	Are the setting, location, and relevant dates provided?	Were participants fairly selected?	Are participant characteristics provided?	Are the measures of exposures and outcomes appropriate?	Is there a description of how the study size was arrived at?	Is there information on how missing data was handled and whether sources of bias were controlled for?	Is information provided on participant eligibility?	Are the results well described?
Cimolin et al (2022) [24]	✓	✓	✓	✓	✓	X	X	X	X
Kaur et al (2023) [25]	✓	✓	Not reported	✓	✓	X	X	X	✓
Khan et al (2021) [26]	✓	✓	Not reported	✓	✓	X	X	X	X
Khan et al (2013) [27]	X	✓	Not reported	X	✓	X	X	X	X
Kondragunta et al (2020) [19]	X	✓	Not reported	X	✓	X	X	X	X
Lai et al (2022) [28]	✓	✓	✓	✓	✓	X	X	X	✓
Li et al (2018) [29]	X	✓	Not reported	X	✓	X	X	X	✓
Mehdizadeh et al (2021) [9]	✓	✓	✓	✓	✓	X	X	✓	✓
Mehdizadeh et al (2021) [15]	✓	✓	✓	✓	✓	✓	X	✓	✓
Morinan et al (2022) [30]	✓	✓	X	X	✓	X	X	X	X
Muñoz-Ospina et al (2022) [31]	✓	✓	Not reported	✓	✓	X	X	X	✓
Ng et al (2020) [16]	✓	✓	✓	✓	✓	X	X	X	✓
Ospina et al (2021) [32]	✓	✓	✓	✓	✓	X	X	X	✓
Otte et al (2020) [33]	✓	✓	✓	✓	✓	✓	✓	X	✓
Pedro et al (2020) [34]	X	X	Not reported	✓	✓	X	X	X	X
Procházka et al (2015) [35]	X	X	Not reported	X	✓	X	X	X	✓
Rupprechter et al (2021) [36]	✓	X	Not reported	X	✓	X	X	X	✓
Sabo et al (2022) [18]	✓	✓	✓	✓	✓	X	X	X	✓
Sabo et al (2022) [37]	✓	✓	Not reported	✓	✓	X	X	X	✓
Sabo et al (2021) [17]	✓	✓	Not reported	X	✓	X	X	X	X
Sabo et al (2020) [38]	✓	X	Not reported	✓	✓	X	X	X	✓
Seifallahi et al (2022) [39]	✓	X	Not reported	X	✓	X	X	X	✓
Shin et al (2021) [40]	✓	✓	✓	✓	✓	X	X	X	✓
Soltaninejad et al (2018) [41]	X	✓	Not reported	X	✓	X	X	X	✓
Tan et al (2019) [42]	✓	X	✓	✓	✓	✓	X	X	✓
Ťupa et al (2015) [43]	✓	✓	Not reported	X	✓	X	X	X	✓

### Patient Groups

The 26 studies comprised 18 involving participants with PD, 6 involving participants with dementia, and 2 involving participants with MCI. Most (18/26, 69%) of the studies used Kinect sensors for MMC. All studies that included participants with dementia (n=6) used the Kinect sensor and were conducted in inpatient settings. The Kinect was used to quantify gait decline over 10 weeks [9], propose a prognostic model for fall risk [15], and demonstrate the association with clinical gait measures and future falls [16]. In inpatients with dementia and drug-induced Parkinsonism, the Kinect was used to capture Parkinsonian traits [17]; quantify Parkinsonian gait [38]; and along with pose estimation of recordings from a video camera, estimate Parkinsonian severity [18]. It was also used in the MCI studies reviewed (n=2), as a tool to detect MCI from gait features [19,39].

Of the 18 studies that included participants with PD, 10 (56%) reported the use of the Kinect sensor for analyzing gait, including its feasibility to extract relevant features [34,42], ability to detect PD [24,32,41,43], and ability to measure clinical disease severity [28,31,33]. Alternative MMC models that use image processing for pose estimation of videos from RGB cameras have also been used with participants with PD, demonstrating the feasibility of these models in quantifying gait impairment and disease severity [18,25-27,29,30,36,40].

### Functional Performance Components

Most studies (24/26, 92%) evaluated aspects of gait, although there were significant variations in the features extracted and methods used for analysis, with some of them lacking statistical significance. Other functional performance components evaluated were sit to stand (n=5) and stepping in place (n=1). Table 2 shows functional performance components by study.

**Table 2:** Patient groups, functional performance components, and feature extraction categories.

Study	Patient group	Functional performance component	Feature category
Cimolin et al (2022) [24]	PD^a^	Gait	Spatiotemporal and stability
Kaur et al (2023) [25]	PD	Gait	Spatiotemporal and symmetry
Khan et al (2021) [26]	PD	Gait	Spatiotemporal
Khan et al (2013) [27]	PD	Gait	Gait posture and spatiotemporal
Kondragunta et al (2020) [19]	MCI^b^	Gait	Spatiotemporal
Lai et al (2022) [28]	PD	Gait	Spatiotemporal and ROM^c^
Li et al (2018) [29]	PD	Sit to stand and gait	Spatiotemporal
Mehdizadeh et al (2021) [9]	Dementia	Gait	Spatiotemporal, ROM, and stability
Mehdizadeh et al (2021) [15]	Dementia	Gait	Spatiotemporal, stability, and symmetry
Morinan et al (2022) [30]	PD	Sit to stand	Spatiotemporal
Muñoz-Ospina et al (2022) [31]	PD	Gait	Spatiotemporal and symmetry
Ng et al (2020) [16]	Dementia	Gait	Stability
Ospina et al (2021) [32]	PD	Gait	Spatiotemporal and symmetry
Otte et al (2020) [33]	PD	Stepping in place	Spatiotemporal, symmetry, and rhythmicity
Pedro et al (2020) [34]	PD	Gait	Spatiotemporal
Procházka et al (2015) [35]	PD	Gait	Spatiotemporal
Rupprechter et al (2021) [36]	PD	Gait	Spatiotemporal
Sabo et al (2022) [37]	PD	Gait	Spatiotemporal
Sabo et al (2022) [18]	Dementia	Gait	Spatiotemporal, stability, and symmetry
Sabo et al (2021) [17]	Dementia	Gait	Spatiotemporal, stability, and symmetry
Sabo et al (2020) [38]	Dementia	Gait	Spatiotemporal, stability, and symmetry
Seifallahi et al (2022) [39]	MCI	Gait	Spatiotemporal
Shin et al (2021) [40]	PD	Gait and sit to stand	Spatiotemporal
Soltaninejad et al (2018) [41]	PD	Gait and sit to stand	Spatiotemporal
Tan et al (2019) [42]	PD	Gait and sit to stand	Spatiotemporal and stability
Ťupa et al (2015) [43]	PD	Gait	Spatiotemporal

^a^PD: Parkinson disease.

^b^MCI: mild cognitive impairment.

^c^ROM: range of motion.

### Feature Categories

Table 2 shows extraction feature categories by study. Spatiotemporal features of gait that were reported as having statistical significance included spatial parameters, such as step length (n=8), step width (n=5), and stride length (n=4), and temporal parameters, such as cadence (n=5), gait velocity (n=4), step time (n=4), stance duration (n=1), double support duration (n=1), stride time (n=1), turning time (n=1), turning speed (n=1), swing time (n=1), step velocity (n=1), and stride velocity (n=1). Other extracted feature categories included symmetry (n=9), stability (n=8), range of motion (n=2), and rhythmicity (n=1).

### MMC Devices and Feature Extraction Methods

Table 3 shows that most studies (18/26, 69%) used Kinect depth cameras (4 used V1; 12 used V2; 1 used Kinect eMotion; and the latest version, the Azure Kinect, was used in 1 study), while the remainder used regular video or mobile phone cameras. Common camera positioning included frontal views (n=4), ceiling-mounted (n=4), and multiple cameras from different angles (n=3). However, camera position was not reported in 7 of the study papers. The majority of studies have developed their own custom programs (n=10, 38%) or have used open-source libraries (n=8, 31%) to identify bodies in frame and extract movements. The use of propriety software was less common (n=5).

**Table 3:** Markerless motion capture devices and feature extraction methods used.

Study	Device (camera or sensor)	Devices, n	Frames per second (fps or Hz)	Position of cameras	Extraction methods
Cimolin et al (2022) [24]	Kinect V2	1	30	Tripod in front	Custom algorithm
Kaur et al (2023) [25]	Video camera	2	30	Front and right side	OpenPose
Khan et al (2021) [26]	Video camera	1	25	Front	Custom algorithm
Khan et al (2013) [27]	Video camera	1	5	Not reported	Custom algorithm
Kondragunta et al (2020) [19]	Kinect V2	1	20	Not reported	OpenPose
Lai et al (2022) [28]	Kinect V2	Not reported	30	Not reported	GaitBEST (LongGood Meditech)
Li et al (2018) [29]	Video camera	1	25	Not reported	Iterative Error Feedback and OpenPose
Mehdizadeh et al (2021) [9]	Kinect V2	1	Not reported	Ceiling in hallway	Custom algorithm
Mehdizadeh et al (2021) [15]	Kinect V2	1	Not reported	Ceiling in hallway	Custom algorithm
Morinan et al (2022) [30]	Mobile phone camera and KELVIN-PD (Machine Medicine) mobile app	Not reported	Not reported	Not reported	OpenPose
Munoz-Ospina et al (2022) [31]	Kinect eMotion	1	Not reported	Not reported	Custom algorithm
Ng et al (2020) [16]	Kinect V2	1	30	Ceiling at the end of a hallway	OpenPose
Ospina et al (2021) [32]	Kinect V1	1	Not reported	Participants walking toward the camera	Custom algorithm
Otte et al (2020) [33]	Kinect V1	1	30	1.4 m height in front	Custom algorithm
Pedro et al (2020) [34]	Azure Kinect	3	30	Each end of walkway and halfway between	Azure Kinect SDK to extract joint positions to estimate 32 body joint poses from depth color recordings
Procházka et al (2015) [35]	Kinect V1	1	30	60 cm above floor	Custom algorithm
Rupprechter et al (2021) [36]	Mobile phone camera and KELVIN-PD (Machine Medicine) mobile app	1	Not reported	Patients walking directly toward or away from the camera in hallways or office settings	OpenPose
Sabo et al (2022) [37]	Logitech C920	1	30	Tripod mounted, at one end of walkway	AlphaPose (Shanghai Jiao Tong University), Detectron (Facebook AI Research), OpenPose, and ROMP (Regress All Meshes in a One-Stage Fashion for Multiple 3D People; JD AI research)
Sabo et al (2022) [18]	Kinect V2 and mobile phone cameras	1	30	Kinect: hallway ceiling; stationary mobile phone camera: participants walked toward and away from	OpenPose, Detectron, and AlphaPose
Sabo et al (2021) [17]	Kinect V2	1	30	Ceiling in hallway	AlphaPose and engineered 2D gait features from joint trajectories
Sabo et al (2020) [38]	Kinect V2	1	30	Ceiling in hallway	OpenPose
Seifallahi et al (2022) [39]	Kinect V2	1	Not reported	On a tripod at a suitable distance from an oval path	Custom algorithm
Shin et al (2021) [40]	Video camera	1	30	Frontal view from a tripod-mounted camera 1.5 m from the horizontal line of the turning point	OpenPose, OpenCV
Soltaninejad et al (2018) [41]	Kinect V2	Not reported	30	Not reported	Graph model of body skeleton
Tan et al (2019) [42]	Kinect V2	1	Not reported	End of walkway	Custom algorithm
Ťupa et al (2015) [43]	Kinect V1	1	30	60 cm above floor	Custom algorithm

### Key Findings

Tables 4-6 summarize the key findings of the 26 studies that used MMC to study movement features in people with dementia, MCI, and PD. Stride length, cadence, gait stability, step length, arm swing, and number of steps were the primary features investigated in these studies. Notably, several studies [26,28,33,37] found that stride length and cadence are commonly affected in those with PD. Other studies [15,31,38] highlighted the potential of MMC for predicting fall risk and discriminating between individuals with PD and controls.

Most studies (20/26, 77%) used some form of clinical validation for the assessment of disease, and patients were referred to or assessed within a clinical research facility by a clinician. The most common clinical measures used were the Unified Parkinson’s Disease Rating Scale (UPDRS) for assessing Parkinsonism symptoms in those with PD and dementia and the Performance Oriented Mobility Assessment–gait and Performance Oriented Mobility Assessment–balance assessments for evaluating mobility characteristics. Many of the studies (23/26, 88%) used the MMC features to classify patients from control participants and to classify symptom severity (eg, UPDRS scores in PD) using various techniques, including support vector machines, random forest models, multivariate ordinal logistic regression, and adaptive neuro-fuzzy inference system classifiers. Several studies reported excellent classification accuracy, with some achieving 100% accuracy [26,27,41]. For instance, Seifallahi et al [39] achieved an accuracy of over 90% for differentiating between people with MCI and controls using an adaptive neuro-fuzzy inference system classifier. Khan et al [26] reported a 70.83% accuracy in predicting UPDRS-gait scores using a support vector machine model, with an area under the receiver operating characteristic curve of 80.88%.

Conversely, most studies included within this review (20/26, 77%) did not evaluate their MMC system or algorithms against an established gold-standard motion capture model, making it difficult to conclude whether their derived features for monitoring functional performance characteristics were comparable to an accepted measure of movement analysis. Some notable exceptions such as Cimolin et al [24] compared their Kinect setup to a Vicon system, which is an accepted and clinically validated method for assessing gait. Other studies used established and clinically validated spatiotemporal measures including the GAITRite system [34,40] and the Zeno Walkway system [37], although the study by Pedro et al [34] only had 2 participants. Li et al [29] had experts manually annotate videos, which, while subjective, proved effective for creating labels to train machine learning algorithms for task segmentation. They also used automated labeling to generate subtask segmentation, which could help automate larger-scale studies and clinical assessments.

MMC models showed moderate to strong positive correlations with Vicon [24], Zeno [37], and GAITRite [40]. However, some of the studies also identified limitations of MMC. For example, Pedro et al [34] found that Kinect cameras may overestimate step length variation in people with PD due to inherent smoothing, while Sabo et al [37] found that automated heel strike algorithms may struggle to identify short steps. Some studies [19,29] reported challenges with data processing and interpretation, highlighting the need for more standardized methods in this field.

Despite these limitations, the findings suggest that MMC is a promising tool for studying characteristics of functional performance in people with dementia, MCI, and PD. It is worth noting that specialized depth cameras may not be necessary for extracting suitable joint positions in camera space [37]. However, further research in this field is warranted to fully understand the potential of MMC.

**Table 4:** Key findings from studies that used the Kinect.

Study	Primary features	Main results
Cimolin et al (2022) [24]	Gait cadence, mediolateral sway, and step width	Strong positive correlation between Kinect and Vicon systems for gait cadence and mediolateral sway (ICC^a^ 0.94-0.97) and a weak correlation for step width (ICC 0.44) in people with PD^b^
Kondragunta et al (2020) [19]	Gait cycle (dynamic time warping)	SVM^c^ for classifying between controls, persons with possible MCI^d^, and persons with MCI: 74.6%-87.3%
Lai et al (2022) [28]	Stride length, straight walking speed, and turning speed	Mediation analysis demonstrates decreased stride length, walking speed, and turning speed are associated with increased falls prediction model score (*r*=–0.58, *r*=–0.52, and *r*=–0.46, respectively; *P*<.001) UPDRS^e^ negatively correlated with features (*r*=–0.65, *r*=–0.56, and *r*=–0.37, respectively; *P*<.001) but positively with fall prediction model score (*r*=.53, *P*<.001) UPDRS serves as a mediator for features and higher fall prediction model scores
Mehdizadeh et al (2021) [9]	Gait stability, step time, step length, step time variability, and step length variability	Mixed effects models over 10 weeks show: Decrease in primary features and an increase in variability over time for people with dementia Gait stability decreased more in men Mediolateral range of motion decreased in those with mild neuropsychiatric symptoms but increased in those with more severe symptoms
Mehdizadeh et al (2021) [15]	Gait stability.	Cox proportional hazard regressions show gait stability predicts time to fall in people with dementia (ROC^f^ 0.80 at 7 days, 0.67 at 30 days)
Muñoz-Ospina et al (2022) [31]	Left and right arm and ankle swing (magnitude and speed), stance time, gait speed, total time, and number of steps	Random forest model was most accurate for discriminating between people with PD and controls (85% using all gait features)
Ng et al (2020) [16]	Gait: cadence, symmetry, CV^g^ of step time, step width (average and CV), and eMOS^h^	Univariate linear regression: cadence associated with POMA^i^-gait scores (*P*<.001) Poisson regression: cadence, eMOS, average step width associated with the number of future falls (*P*<.001)
Ospina et al (2021) [32]	Arm swing: magnitude, time, and arm swing asymmetry	Age influenced arm movement People with PD showed significant reductions in arm swing magnitude (left, *P*=.002; right, *P*=.006) and speed (left, *P*=.002; right, *P*=.004) Arm swing asymmetry differentiated people living with PD from controls (ROC: 78%)
Otte et al (2020) [33]	Cadence, knee amplitude, asymmetry, average step time, longest step time, arrhythmicity, average stance time, and longest stance time	Knee amplitude and longest stance time correlated with UPDRS (–0.51, *P*=.003 and 0.52, *P*=.002, respectively) Postural instability (pull test) correlated with longest stance time (0.47, *P*=.008) Knee amplitude, asymmetry, and average step time differed between on- and off-medication states (*P*=.002, *P*=.007, and *P*=.007, respectively)
Pedro et al (2020) [34]	Step length	In comparison with the GAITRite (CIR Systems, Inc) system, the Kinect camera overestimated the average variation in step length for the 2 people with PD potentially due to inherent smoothing
Procházka et al (2015) [35]	Average step length	In total, 91.7% classification accuracy for determining between controls and those with people with PD. Decrease in step length (regression coefficient=–0.0082 m/year)
Sabo et al (2022) [18]	Number of steps, cadence, velocity, step length, CV of stride width, and step and swing time	Moderate or strong positive correlations between steps, cadence, step width from 2D pose-estimation, and Zeno in people with PD Automated heel strike algorithm struggled to identify short steps
Sabo et al (2021) [17]	Cadence, steps, average step width, average margin of stability, CV of step width and time, and symmetry	ST-GCN^j^ using 2D joint trajectories and gait features outperforms ST-GCN using only gait features Regression models for predicting UPDRS-gait over 94% if off by 1 is allowed
Sabo et al (2020) [38]	2D: steps, cadence, symmetry, and CV of step time 3D: walking speed, step length or width, step width, step length symmetry angle, RMS^k^ of ML^l^ velocity, margin of stability, and CV step width	Multivariate ordinal logistic regression models achieved 61.4% and 62.1% for 2D and 3D features for predicting UPDRS-gait in people with dementia
Seifallahi et al (2022) [39]	Steps and stride	Adaptive neuro-fuzzy inference system classifier accuracy >90% for differentiating between MCI and controls
Soltaninejad et al (2018) [41]	Stride and tremor	Random forest classifier accuracy for differentiating controls and people with dementia: 93.33% stride and 81% tremor
Tan et al (2019) [42]	Step length, step time, vertical pelvic displacement, and gait speed	Multivariable regression: step length during TUG^m^ and vertical pelvic displacement during the gait speed were associated with postural instability and gait disorder (*P*=.01 and *P*<.05, respectively) in people with PD
Ťupa et al (2015) [43]	Step length and average speed	Combining gait features improves classification accuracy relative to single features 2-layer neural network achieved an accuracy of 97.2% in classifying people with PD from controls

^a^ICC: intraclass correlation coefficient.

^b^PD: Parkinson disease.

^c^SVM: support vector machine.

^d^MCI: mild cognitive impairment.

^e^UPDRS: Unified Parkinson’s Disease Rating Scale.

^f^ROC: receiver operating characteristic.

^g^CV: coefficient of variation.

^h^eMOS: estimated margin of stability.

^i^POMA: Tinetti Performance Oriented Mobility Assessment.

^j^ST-GCN: spatiotemporal graph convolutional networks.

^k^RMS: root mean squared.

^l^ML: mediolateral.

^m^TUG: Timed Up and Go.

**Table 5:** Key findings from studies that used video cameras.

Study	Primary features	Main results
Kaur et al (2023) [25]	Stride (91 derived features based on variation and asymmetry speed)	Logistic regression, random forest, deep learning–based classifiers 75% (walking and talking) and 78.1% (walking) Multi-scale residual neural network: 100% accuracy for classifying people with controls, multiple sclerosis, and people with PD^a^ during walking and walking-while-talking, and 78% for new subjects walking 1D convolutional neural network: 75% walking-while-talking and 79.3% when generalizing to new subjects in different tasks
Khan et al (2021) [26]	Slow walking short-shuffling steps gait festination	SVM^b^ classification predicts UPDRS^c^: gait scores with 70.83% accuracy and area under ROC^d^ curve 80.88%
Khan et al (2013) [27]	Stride cycles and posture lean	SVM classification of 100% for differentiating between people with PD and controls
Li et al (2018) [29]	Subtask segmentation based on selected body points: neck, R/L^e^ shoulder, R/L hip, R/L knee, or R/L ankle	Accuracies for subtask segmentation of TUGg: OpenPose+LSTM^f^=93.10% and OpenPose+LSTM=92.8% Correlations between OpenPose+LSTM and experts on timed reduction rates: turn (0.93), walk-back (0.98), and sit-back (0.98)
Sabo et al (2022) [37]	Cadence, steps, average step width, average margin of stability, CV^g^ of step width and time, symmetry, and stability	ST-GCN^h^ operating on 3D joint trajectories outperform 2D models Best model prediction of UPDRS-gait and SAS^i^-gait scores are 53% and 40%, respectively.
Shin et al (2021) [40]	Step length, gait velocity, number of steps, and turning time	Features correlated with Freezing of Gait Questionnaire, UPDRS part III total score, HY^j^, and postural instability in people with PD Features measured improvements following medication

^a^PD: Parkinson disease.

^b^SVM: support vector machine.

^c^UPDRS: Unified Parkinson’s Disease Rating Scale.

^d^ROC: receiver operating characteristic.

^e^R/L: right or left.

^f^LSTM: long short-term memory (machine learning model).

^g^CV: coefficient of variation.

^h^ST-GCN: spatiotemporal graph convolutional networks.

^i^SAS: Simpson-Angus Scale.

^j^HY: Hoehn and Yahr scale.

**Table 6:** Key findings from studies that used mobile phone cameras.

Study	Primary features	Main results
Morinan et al (2022) [30]	*D_body_*: distance between nose and 2 ankles Standard of *D_body_*, proportional increase in *D_body_*, and percentage jerk of *D_body_* *D_hand_*: distance between 2 wrists U: hands used (Boolean)	Ordinal random forest classifiers: U=99.6% accuracy for hands used to push up from chair UPDRS^a^ ratings estimated by models agree by 79.2% with clinicians’ ratings for people with PD^b^
Rupprechter et al (2021) [36]	Steps, arm swing, postural control, and smoothness	Step frequency highly correlated with labeled steps (*P*<.001) Ordinal random forest: 50% prediction

^a^UPDRS: Unified Parkinson’s Disease Rating Scale.

^b^PD: Parkinson disease.

## Discussion

### Principal Findings

This systematic review has shown that there is a paucity of studies exploring the use of MMC in people with dementia and models exploring the performance of ADL. Moreover, there is a lack of standardization in the used MMC models and clinical validation in real-world applications. The absence of standardization among the models used posed a significant challenge, precluding the possibility of conducting a meta-analysis to compare and synthesize study results.

The review findings suggest that there is more evidence of the use of MMC with full-body tracking in patients with PD (n=18) compared to those with dementia (n=6) and MCI (n=2). This demonstrates a bias toward movement disorders, where the motor symptoms are more prominent, and highlights a significant knowledge gap in the feasibility and effectiveness of using MMC models in quantifying functional performance in people with dementia and MCI. Moreover, the studies that included patients with dementia [9,15-18,38] were all conducted in inpatient dementia units, indicating a lack of research involving this patient group in real-world settings. This underscores the need for further investigation in this area.

While MMC models based on gait features extracted mainly from straight-line walking may provide useful preliminary data for model development, they have less scope in quantifying functional performance in a real-world context, particularly in people with cognitive impairment. In contrast, the evaluation of ADL tasks could potentially provide more comprehensive insights into real-world functional performance from routine daily activities. Previous research suggests that dual-task tests of mobility are more effective in detecting cognitive decline as well as predicting cognitive impairment and falls [44-46], potentially due to the increased cognitive demand on the individual. However, just 1 study included in this review [19] used dual tasks for the classification of MCI from control, and it was not reported how the completion of dual tasks impacted the results. Feature extraction of ADL tasks that require planning and organization could potentially facilitate the measurement of dual-task performance. Therefore, analysis of ADL tasks could help provide a more accurate assessment of neurodegenerative impairment.

The findings of this review suggest a lack of consensus on the most effective features used. Some spatiotemporal features of mobility such as step length are commonly used, but other features vary widely between studies, making it difficult to determine which are most effective. Additionally, some unique features such as vertical pelvic displacement [42] and *D_body_,* the distance between nose and 2 ankles [30], have been identified in individual studies, but their effectiveness is unknown without further evaluation. Moreover, it is important to note that the effectiveness of several of these feature extraction models has not been tested in real-world settings which therefore requires further evaluation.

Several studies included in the review (n=10) reported machine learning classifier outcomes for identifying people living with dementia, MCI, or PD from control [19,25-27,31,32,35,39,41,43], whereas several others (n=8) reported models that computed clinical assessment scores [17,18,29,30,33,36,38,40]. Although these are useful outcomes, it is important to note that models that help detect gait impairment and predict falls (n=5) [9,15,16,28,42] could potentially be more useful in practical applications for assessing functional performance. It must also be noted that these models were all based on the Kinect cameras demonstrating the potential of RGB-D cameras for detecting and predicting functional impairment.

Accurate feature extraction and classification are crucial for improving the quality of MMC-based functional assessment [47]. The accuracy rates of MMC models reported in the reviewed studies ranged from 40% for a model predicting a clinical assessment score [18] to 100% for machine learning classification of PD from control [27]. Those numbers cannot be compared directly due to the different number of classes and the resulting chance level, as well as the task difficulty between classifying patients from control participants to rating symptoms. However, it is important to ensure that any clinical applications of these models are consistent and accurate because inaccurate predictions could potentially have consequences for patient care. Further validation and refinement of the models may therefore be necessary before they can be safely used in practical applications.

It is important to note that the accuracy of a model does not only depend on its ability to correctly identify a condition but also on its capacity to detect features of functional performance consistently in various real-world settings. Potential real-world applications include the detection of problems in functional performance in clinical settings and functional deterioration in home settings. The effectiveness of several feature extraction models reviewed in this study has not been tested in such settings, and therefore, the accuracy in practical applications remains unclear. Moreover, devices used in clinical applications must be subjected to a rigorous clinical validation process to ensure safety and efficacy before use on patients [48]. Many of the studies reviewed seem to have primarily focused on the technical aspects of the MMC models, such as feature extraction and analysis, with less focus on their clinical utility. Therefore, further MMC research should objectively evaluate the practical clinical and real-world mobility applications of this technology. If a standard MMC movement analysis protocol could be established, functional performance could be compared across diagnoses.

Additionally, the cross-sectional nature of most of the included studies may limit their ability to evaluate and track functional performance over time. Longitudinal studies would be necessary to assess the performance of these models for tracking functional changes caused by factors such as disease progression, infections, and treatment effects or recovery. Despite these limitations, the effectiveness of MMC models using the Kinect [24] and 2D pose estimation [37] in comparison to established gold-standard motion capture systems within experimental settings suggests they may be suitable for testing in real-world applications such as remote monitoring. However, further research is required to explore and address ethical and privacy considerations when deploying MMC devices that capture video and movement within people’s homes. Managing consent where patients lack mental capacity and safeguarding the privacy of patient data that is stored or shared with clinical teams will also need to be carefully addressed while deploying MMC in remote monitoring applications.

It is important to consider the overall quality of studies included in this review, as shown in Table 1, which summarizes key questions to consider when assessing quality. Most studies had a clear study design and focused research questions with appropriate measures of exposures and outcomes. However, only 1 of the studies reported if potential sources of bias from confounders such as musculoskeletal comorbidities, were controlled which could have significant implications for clinical applications. Moreover, few studies provided information on participant eligibility and whether they were selected fairly which could have implications for generalization of study results. The suboptimal quality observed in the included studies in key aspects such as bias control and participant selection suggests these MMC models need to be further evaluated potentially using more rigorous study designs before deployment in real-world applications. Studies that have attempted to create MMC models for fall prediction have primarily focused on retrospective analyses, for example, the number of falls in the past few months. While it is useful to examine historical patterns, future studies should aim to develop prospective studies. Testing the algorithms for MMC models in a prospective study would offer the capability to analyze more detailed information on fall events and contextual associated factors therefore making them more generalizable and valid for predicting falls.

The main findings of this review highlight the potential of MMC in assessing components of functional performance including gait and sit-to-stand characteristics in individuals with dementia, MCI, and PD. Notably, high classification accuracies in several studies demonstrate the potential for clinical applications, such as identifying, monitoring, and predicting outcomes in these populations. However, it is crucial to address the limitations and challenges, such as overestimation of step length variation and difficulty in identifying short steps, as well as the need for standardized methodologies and further research.

A segment of motion analysis research will likely continue to focus on simplified, discrete tasks executed within the controlled setting of a laboratory. However, advancements in technology are progressively enabling the expansion of movement analysis into real-world environments [49]. While the current body of literature predominantly centers on gait analysis, the potential applications of MMC extend far beyond this domain, particularly within the realm of ADL. The integration of knowledge gleaned from analyzing various types of functional tasks will empower clinicians to better assist individuals with neurodegeneration in enhancing their quality of life.

### Limitations

It was not feasible to conduct a meta-analysis of the reviewed studies due to significant heterogeneity in the MMC models evaluated, the features extracted, and the analysis methods used. The use of search terms that are not specific to MMC such as motion capture and movement analysis may have introduced the possibility of inherent biases in the search results. However, the adoption of these broad search terms facilitated a more comprehensive screening of studies, encompassing a wider spectrum of the literature. Furthermore, it is important to acknowledge a potential constraint inherent in the search strategy, specifically about the inclusion criterion of full-body tracking MMC models. This led to the exclusion of studies that analyzed the movement of specific body parts. Another limitation of this review is that only a small number of studies met the inclusion criteria limiting the generalizability of this study’s results.

### Conclusion

The findings of this review illustrate that the use of MMC technology with full-body tracking has the potential to quantify functional performance in people living with dementia, MCI, and PD. However, the lack of consistency in evaluating these models presents a challenge. Standardization of the extracted features and analysis methods may help overcome the heterogeneity of the evaluation process and propose a framework for assessing future models. The findings further suggest that MMC models based on both RGB-D and standard video cameras are viable options for analyzing movement, yielding similar outcomes. Nonetheless, RGB-D cameras have been favored in models intended to detect gait impairment and predict instances of falling.

It is worth noting that the majority of the reviewed studies evaluated aspects of gait, with no evidence of ADL tasks being analyzed. Future studies should incorporate ADL tasks, as this would be more representative of real-world scenarios, particularly for individuals with cognitive impairment. Moreover, longitudinal studies are required to develop models that could track functional impairment over time and potentially predict decline.

Although accuracy is an important factor to consider when evaluating MMC models for clinical applications, other factors such as comparability to established gold-standard motion capture models and capability for analyzing routine tasks and reproducibility in the natural environment are also important. Therefore, a more holistic approach to model development and evaluation with a clear focus on real-world clinical utility may be necessary to ensure that the models are suitable for use in practical applications.

## Appendix

Multimedia Appendix 1 Search activity record.

## Abbreviations

ADL: activities of daily living
MCI: mild cognitive impairment
MMC: markerless motion capture
PD: Parkinson disease
PRISMA: Preferred Reporting Items for Systematic Reviews and Meta-Analyses
RGB: red-green-blue
RGB-D: red-green-blue+depth
UPDRS: Unified Parkinson’s Disease Rating Scale

## Data Availability

No new or unpublished data are included within this study and all data are freely available. All code relating to summary figure development is available on request to the corresponding authors.
