# Supplementary material for: Markerless Motion Capture to Quantify Functional Performance in Neurodegeneration: Systematic Review
Source: JMIR Aging. 2024 Aug 6;7:e52582. doi: 10.2196/52582 (PMC11336506; doi:10.2196/52582)
Supplement: Multimedia Appendix 1 [file aging_v7i1e52582_app1.docx]

# Search activity

| Research topic: | Markerless motion capture in people living with Dementia, Mild Cognitive Impairment (MCI) and Parkinson’s disease (PD) | | |  |
| --- | --- | --- | --- | --- |
| Places to search for information: | Embase, MEDLINE, CINAHL, and Scopus | | |  |
|  | | | |  |
| **Databases searched:** | **Date of search** | **Search terms used** | **Initial total of results found** | **Merged total (duplicates removed)** |
| EMBASE | 2/11/2022 | Motion capture and dementia; Motion capture and MCI;  Motion capture and PD | 206 | 744 (270) |
|  | 17/11/2022 | Motion analysis and dementia; Motion analysis and MCI;  Motion analysis and PD  Movement analysis and dementia; Movement analysis and MCI; Movement analysis and PD | 774 |  |
|  | 27/1/2023 | Pose estimation and dementia; Pose estimation and MCI;  Pose estimation and Parkinson’s disease | 34 |  |
| MEDLINE | 8/11/2022 | Motion capture and dementia; Motion capture and MCI;  Motion capture and PD | 151 | 304 (107) |
|  | 17/11/2022 | Motion analysis and dementia; Motion analysis and MCI;  Motion analysis and PD  Movement analysis and dementia; Movement analysis and MCI; Movement analysis and PD | 241 |  |
|  | 27/1/2023 | Pose estimation and dementia; Pose estimation and MCI;  Pose estimation and Parkinson’s disease | 19 |  |
| CINAHL | 10/11/2022 | Motion capture and dementia; Motion capture and MCI;  Motion capture and PD | 62 | 200 (117) |
|  | 10/11/2022 | Motion analysis and dementia; Motion analysis and MCI  Motion analysis and PD  Movement analysis and dementia; Movement analysis and MCI; Movement analysis and PD | 255 |  |
|  | 27/1/2023 | Pose estimation and dementia; Pose estimation and MCI;  Pose estimation and Parkinson’s disease | 3 |  |
| Scopus | 15/11/2022 | Motion capture and dementia; Motion capture and MCI;  Motion capture and PD | 235 | 347 (379) |
|  | 15/11/2022 | Motion analysis and dementia; Motion analysis and MCI;  Motion analysis and PD  Movement analysis and dementia; Movement analysis and MCI; Movement analysis and PD | 435 |  |
|  | 27/1/2023 | Pose estimation and dementia; Pose estimation and MCI;  Pose estimation and Parkinson’s disease | 48 |  |
| Grand total | | | | 1595 |
